# Editorial: Psychological interventions addressed to higher education students in student psychological services

**DOI:** 10.3389/fpsyg.2023.1129697

**Published:** 2023-01-23

**Authors:** Barbara Giangrasso, Man Cheung Chung, Isabella Giulia Franzoi

**Affiliations:** ^1^Department of Health Sciences, School of Psychology, University of Florence, Firenze, Italy; ^2^College of Natural and Health Sciences, Zayed University, Abu Dhabi, United Arab Emirates; ^3^Department of Psychology, University of Turin, Turin, Italy

**Keywords:** university students, distress, psychological interventions, mental health, student psychological services

Academic stress among university students is high (Freire et al., 2019). Concerns about how to adapt to a new environment, school performance, overwork, and thinking about the future are all possible stressors (Beiter et al., 2015), and social, relational, and financial problems affect undergraduates' performance and health (DeRosier et al., 2013). It has been observed that student mental health represents one of the major challenges to global public health (Storrie et al., 2010). When compared with the general population of the same age group, college students show high rates of mental health problem (Keyes and Westerhof, 2012).

It is possible that emerging adults have difficulty recognizing their own abilities and qualities, as well as a defective sense of consistency between others' impressions of them and their own self-perceptions. Students' vulnerability to suicide may be increased by judgment, attack on the self, idealized aspirations, and the fantasy that every choice can alter the course of the future (Granieri et al., 2022).

Counseling services have been implemented in many tertiary education institutions around the world (Kraft, 2009), and student counseling centers have proven to be a unique provider of support for these young people (Gallagher, 2009; Buchanan et al., 2012). Collecting data on student mental health and academic outcomes has significant implications for mental health service on campuses (Farrer et al., 2019).

Such issues are supported by the 8 articles which were included in the Research Topic “Psychological interventions addressed to higher education students in student psychological services”.

A recent study conducted on 671 students of an Italian University showed that medical students exhibit a higher prevalence of suicidal ideation and depression than detectable in the general population (50.2 and 16.7% vs. 15–18% and 9.2%, respectively) and that these clinical variables correlate with some specific parenting styles. It would appear that females experience more distress and self-injurious behaviors, while males apparently seem more disposed to drug use or alcohol abuse (Tugnoli et al.).

Another Italian study showed student's problems in two main areas: the emotion/relationship area (in which problems in emotional dysregulation seem to be associated with different personality pathological domains, such as antagonism, psychoticism, negative affectivity, disinhibition, and detachment) and the area of learning (where the motivational aspects concerning self-confidence, learning goals and academic self-efficacy are differently associated with resilience and anxiety). In the study by Pace et al., the comparison between the pre- and post-COVID period showed that the number of students experiencing high levels of anxiety in the learning area increased.

Interest in the role of positive psychology and positive emotions in the learning process has emerged (e.g., Rand et al., 2011; You and Kang, 2014); students' psychological wellbeing is an important issue in higher education.

To achieve the systematization of intervention protocols aimed at promoting wellbeing, it is important to conduct controlled and randomized studies. An ongoing example is represented by the study proposing the NoiBene protocol (Di Consiglio et al.), a group setting that aims to foster mental health and wellbeing, improve aspects such as assertiveness, emotional awareness, commitment to values, goal setting, and decrease dysfunctional mechanisms such as repetitive thinking, perfectionism, and avoidant experiences. The design involves random assignment of participants to three conditions: the NoiBene group condition (NB-G), the NoiBene web-guided condition (NB-WB) or the waiting list condition (WLC). Pre- and post-intervention data and follow-up at 6 months were analyzed. Indeed, it is through research designs of this kind that we can identify useful pathways for promoting wellbeing (Weiss et al., 2016).

Among the psychological factors that contribute to the mental health and academic performance of university students and increase the chance of employment for these students, academic self-efficacy, psychological wellbeing (Costa et al., 2013), self-confidence, self-esteem, and empowerment (Jaaffar et al., 2019) were found to play important roles.

Self-esteem and self-acceptance are factors that influence mental health. As evidenced by the study by Qian et al., psychoeducational lessons and group interventions on self-esteem and self-acceptance can be effective tools to promote student wellbeing. In particular, the study carried out on Chinese students highlights how the psychoeducational lesson and the intervention focused on oneself are effective approaches to improve the self-esteem of university students, while self-acceptance is promoted more through the intervention self-focused group.

Only about half of tertiary education European institutions offer psychological services to students. The low percentage of studies on this topic may result from variations with respect to the large difference in registration rates between European and non-European countries. Research include findings that, after psychotherapy, students experience increased levels of wellbeing and reduced levels of distress, asserting the viability of psychotherapy in upgrading students' mental health (Franzoi et al.).

Yuan et al. studied the relationship between depression and social avoidance in college students, investigated the mediating roles of trust and loneliness and the regulatory role of self-esteem, to offer a theory-based on inner mechanism intervention approach. The study highlighted that social avoidance not only influences college students' depression directly, but also has an indirect impact through interpersonal trust and loneliness. These traits have causal mediating effects between social avoidance and depression in college students, and self-esteem appears to mediate this process.

Chinese universities have established a psychological crisis counseling intervention system and have begun supervising psychological observers. A study conducted by Zhan and Xia revealed that psychological observers have a more adverse impression of the conduct of suicide interviews with their classmates. The researchers hypothesized this might be attributable to the limited psychological knowledge on the part of psychological monitors. They suggested that professional psychological monitors at universities should therefore provide professional and systematic training.

From a different perspective, the work of Yu and Cai investigated students' experience with online learning using a survey conducted within Chinese universities immediately after COVID-19. They observed that the directness of feedback in using online learning has an impact on students' problem solving skills. This effect of immediacy on intention to use online learning is significant and improves students' problem solving skills (Yu and Cai).

It is important to catalog data on counseling services for students as it allows tertiary education institutions to be encouraged to invest in these services in order to promote mental health. The presence of adequately trained professionals and the ability to offer long-term therapy would allow intercepting health needs for all students who fail to access treatment (Franzoi et al.).

Studies have shown that such interventions maintain their effectiveness over time by improving both students' mental health and academic performance (Monti et al., 2016; Cerutti et al., 2020). In contrast, untreated mental distress can lead to both dropout from academic careers and worsening mental health conditions such as increased suicidal behavior, alcoholism, and drug addiction (Koutra et al., 2010; Pillay, 2021).

Offering psychological support to students allows them to support young adults while providing feedback to the educational institution (Adamo et al., 2012). Although such counseling interventions are often directed at resolving problems that affect academic performance, they can also be an indication for students with stable psychopathology, giving them the opportunity to access mental health services or psychotherapeutic treatment (Biasi et al., 2017).

## Author contributions

All authors listed have made a substantial, direct, and intellectual contribution to the work and approved it for publication.
